# Endoscopic ultrasound-guided caudate lobe liver abscess drainage

**DOI:** 10.1055/a-2174-5604

**Published:** 2023-10-06

**Authors:** Junichi Kaneko, Hiroki Tamakoshi, Moeka Watahiki, Daisuke Kusama, Tomoyuki Niwa, Masaki Takinami, Takanori Yamada

**Affiliations:** Department of Gastroenterology, Iwata City Hospital, Shizuoka, Japan

Endoscopic ultrasound (EUS)-guided liver abscess drainage is an alternative method in cases where percutaneous drainage (PCD) is difficult to perform 1 2 . However, reports on EUS-guided caudate lobe liver abscess drainage remain scarce, with only 12 reported cases 3 . Of these cases, 33 % (4 /12) were treated with a transesophageal approach, which can cause mediastinitis, mediastinal emphysema, and pneumothorax 4 5 . Herein, we present a case of safe and successful drainage via this method using marking clips and adjusting the scope position.

A 50-year-old man with type 2 diabetes mellitus was hospitalized for fever and epigastralgia evolving for 4 days. Computed tomography (CT) revealed a 4.5 × 5.1-cm abscess in the liver caudate lobe ( Fig. 1 ). PCD was not performed because of poor visualization on abdominal ultrasonography. After 2 weeks of antibiotic treatment with little improvement, he was referred to our hospital, where EUS-guided caudate lobe liver abscess drainage was performed ( Video 1 ).

**Fig. 1:**
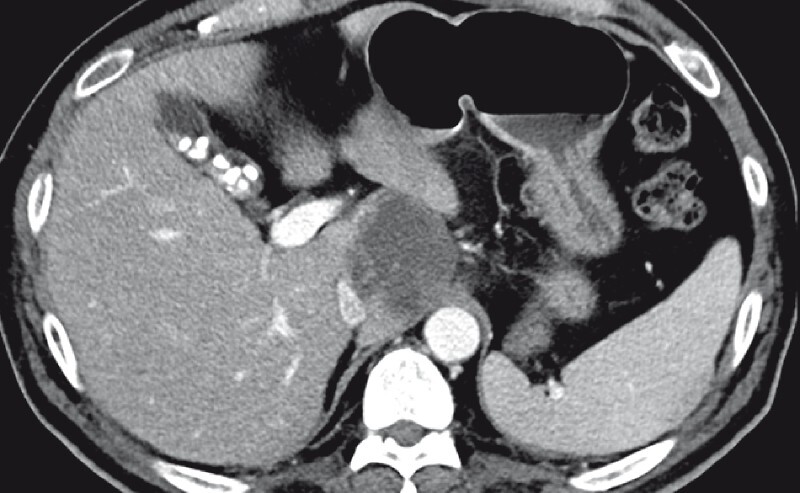
Computed tomography showing a 4.5 × 5.1-cm abscess in the caudate lobe of the liver.

**Video 1** Endoscopic ultrasound-guided caudate lobe liver abscess drainage.


First, a forward-viewing endoscope was used to mark the esophagogastric junction with a clip so that its location could be determined under fluoroscopy. Subsequently, an echoendoscope was introduced into the stomach. By advancing the scope further from the clip and applying an upward angle, the abscess was successfully visualized from within the stomach. The abscess was punctured using a 19-gauge needle with fluoroscopic confirmation of the marking clip, and a 0.025-inch guidewire was inserted into the abscess cavity. Another 0.035-inch guidewire was placed in the abscess cavity using a double-lumen catheter. After dilation using an electrocautery dilator, a 7-Fr double-pigtail plastic stent and a 6-Fr naso-abscess tube were placed ( Fig. 2 ). *Klebsiella pneumoniae* was cultured from the abscess contents ( Fig. 3 ). The nasal tube was removed, and the patient was discharged on day 10 without any complications. The plastic stent was removed 3 months later without liver abscess recurrence.

**Fig. 2:**
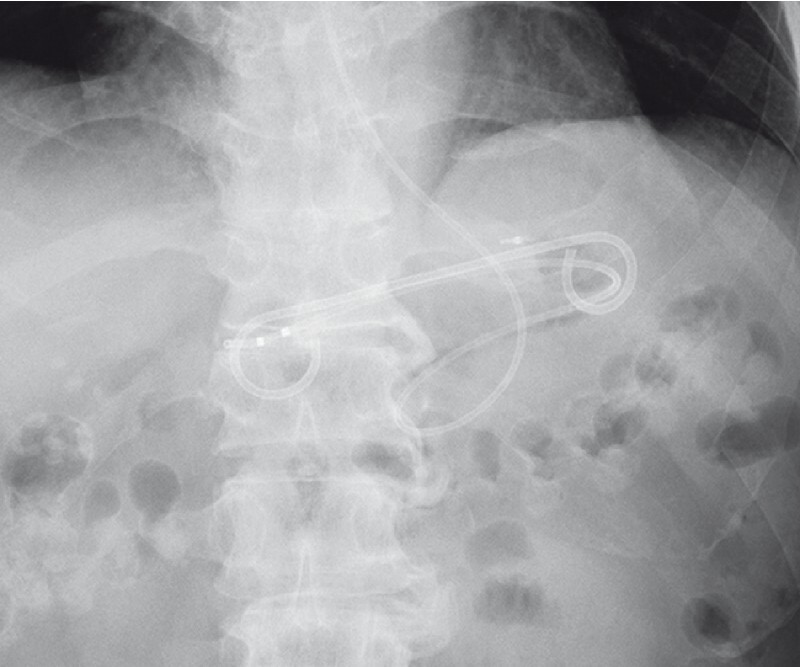
A 7-Fr double-pigtail plastic stent and a 6-Fr naso-abscess tube are visible in the abscess cavity.

**Fig. 3:**
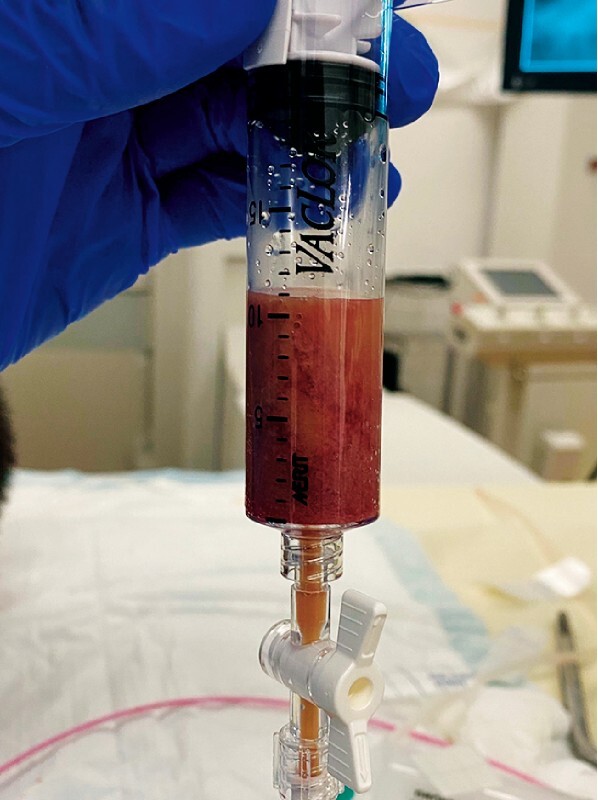
Red-white pus is aspirated through the naso-abscess tube.

Endoscopy_UCTN_Code_TTT_1AS_2AD

## Notes

Endoscopy E-Videos

https://eref.thieme.de/e-videos
*E-Videos* is an open access online section of the journal *Endoscopy* , reporting on interesting cases and new techniques in gastroenterological endoscopy. All papers include a high-quality video and are published with a Creative Commons CC-BY license. Endoscopy E-Videos qualify for HINARI discounts and waivers and eligibility is automatically checked during the submission process. We grant 100% waivers to articles whose corresponding authors are based in Group A countries and 50% waivers to those who are based in Group B countries as classified by Research4Life (see: https://www.research4life.org/access/eligibility/ ). This section has its own submission website at https://mc.manuscriptcentral.com/e-videos
